# NUPR1 is a critical repressor of ferroptosis

**DOI:** 10.1038/s41467-021-20904-2

**Published:** 2021-01-28

**Authors:** Jiao Liu, Xinxin Song, Feimei Kuang, Qiuhong Zhang, Yangchun Xie, Rui Kang, Guido Kroemer, Daolin Tang

**Affiliations:** 1 The Third Affiliated Hospital, Key Laboratory of Protein Modification and Degradation, Guangzhou Medical University, 510600 Guangdong, China; 2 Department of Surgery, UT Southwestern Medical Center, Dallas, TX 75390 USA; 3 Department of Surgery, University of Pittsburgh, Pittsburgh, PA 15219 USA; 4 Department of Oncology, The Second Xiangya Hospital, Central South University, Changsha, Hunan China; 5 Université Paris Descartes, Sorbonne Paris Cité, 75006 Paris, France; 6 Equipe 11 labellisée Ligue Nationale contre le Cancer, Centre de Recherche des Cordeliers, 75006 Paris, France; 7 Institut National de la Santé et de la Recherche Médicale, U1138 Paris, France; 8 Université Pierre et Marie Curie, 75006 Paris, France; 9 Metabolomics and Cell Biology Platforms, Gustave Roussy Cancer Campus, 94800 Villejuif, France; 10 Pôle de Biologie, Hôpital Européen Georges Pompidou, AP-HP, 75015 Paris, France; 11 Department of Women’s and Children’s Health, Karolinska University Hospital, 17176 Stockholm, Sweden

**Keywords:** Cell death, Cell signalling

## Abstract

Ferroptosis is a type of iron-dependent regulated cell death, representing an emerging disease-modulatory mechanism. Transcription factors play multiple roles in ferroptosis, although the key regulator for ferroptosis in iron metabolism remains elusive. Using NanoString technology, we identify NUPR1, a stress-inducible transcription factor, as a driver of ferroptosis resistance. Mechanistically, NUPR1-mediated LCN2 expression blocks ferroptotic cell death through diminishing iron accumulation and subsequent oxidative damage. Consequently, LCN2 depletion mimics NUPR1 deficiency with respect to ferroptosis induction, whereas transfection-enforced re-expression of LCN2 restores resistance to ferroptosis in NUPR1-deficient cells. Pharmacological or genetic blockade of the NUPR1-LCN2 pathway (using *NUPR1* shRNA, *LCN2* shRNA, *pancreas-specific Lcn2 conditional knockout mice*, or the small molecule ZZW-115) increases the activity of the ferroptosis inducer erastin and worsens pancreatitis, in suitable mouse models. These findings suggest a link between NUPR1-regulated iron metabolism and ferroptosis susceptibility.

## Abstract (web summary)

Ferroptosis is an iron-dependent form of oxidative cell death. In this study, the authors show that NUPR1, a stress-inducible transcription factor, may be a driver of ferroptosis resistance.

## Introduction

Cell death is a fundamental physiological process to maintain homeostasis through the removal of supernumerary, unnecessary, or dysfunctional cells, while pathological death can lead to disease. Unlike accidental cell death, regulated cell death follows multiple subroutines, each of which exhibits distinct molecular cascades and regulatory pathways^1,2^. In recent years, an increasing level of interest has been manifested with regard to ferroptosis, a non-apoptotic-regulated cell death^3,4^, which plays a possible pathogenic role in cancer, neurodegeneration, and organ dysfunction^5–7^. The induction of ferroptosis has been shown to rely on iron accumulation, which facilitates oxidative damage through either the production of highly reactive hydroxyl free radicals in the Fenton reaction or the activation of iron-containing enzymes, such as lipoxygenase^8^. Ferroptotic cells exhibit a necrosis-like morphology and damage-associated molecular patterns (DAMPs) released from ferroptotic cells may function as extracellular inflammatory mediators to contribute to tissue injury^9^.

Ferroptosis is regulated at multiple levels, including at the level of transcription factors that may modulate the resistance of malignant cells to anticancer drugs^10^. Such transcription factors do not only participate in rapid responses to ferroptotic stimuli, but also modulate the long-term outcome of ferroptosis in a context-dependent manner^11^. For example, nuclear factor, erythroid 2-like 2 (NFE2L2/NRF2) serves as a master antioxidant transcription factor for blocking ferroptosis^12–14^, whereas the tumor suppressor TP53 plays a dual role in ferroptosis, depending on the tumor type^15–17^. Although many advances have recently been achieved in the comprehension of antioxidant responses and membrane repair mechanisms^18^, the key transcription factor responsible for controlling iron-dependent ferroptosis has been elusive.

Here, we report that the stress response gene, nuclear protein 1, transcriptional regulator (NUPR1) transactivates the gene encoding lipocalin 2 (LCN2) to diminish iron-induced oxidative damage and to induce ferroptosis resistance. Pharmacological or genetic blockade of the NUPR1–LCN2 pathway may enhance the anticancer activity of ferroptosis activator and pathologic inflammation in vitro and in vivo.

## Results

### NUPR1 acts as a repressor of ferroptosis

**Fig. 1:**
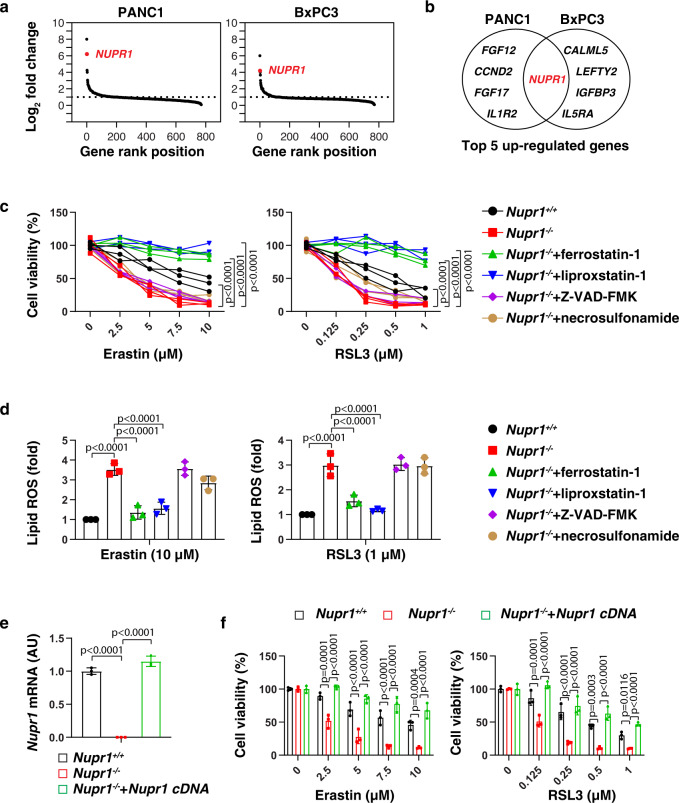
NUPR1 acts as a repressor of ferroptosis. **a** A NanoString technology-based screening of differentially expressed tumor-associated genes in PANC1 and BxPC3 cells following treatment with erastin (10 µM) for 24 h. **b** Top 5 upregulated genes. **c**, **d** *Nupr1*^*+/+*^ and *Nupr1*^−*/*−^ mPDAC cells were treated with erastin or RSL3 in the absence or presence of ferrostatin-1 (1 µM), liproxstatin-1 (1 µM), Z-VAD-FMK (10 µM), or necrosulfonamide (1 μM) for 24 h, and then cell viability (**c**) and lipid ROS (**d**) was measured (*n* = 3 well/group, two-way ANOVA with Tukey’s multiple comparisons test on all pairwise combinations). **e** qPCR analysis of NUPR1 expression in indicated mPDAC cells (*n* = 3 well/group, one-way ANOVA with Tukey’s multiple comparisons test on all pairwise combinations). **f** Cell viability of indicated mPDAC cells following treatment with erastin or RSL3 for 24 h (*n* = 3 well/group, two-way ANOVA with Tukey’s multiple comparisons test on all pairwise combinations). Data in **d**–**f** are presented as mean ± SD. The results in **c**–**f** are representative of those from 2 to 3 independent experiments with three technical replicates each. The results in **a** are representative of those from one independent experiment with three technical replicates each.

A number of small-molecule compounds, including erastin and RSL3, are regularly used to induce ferroptosis and are considered as ‘classical’ inducers of this regulated cell death subroutine^10^. To identify regulators of ferroptosis, we first evaluated the impact of erastin on the expression of 770 tumor-associated genes in two human pancreatic ductal adenocarcinoma (PDAC) cell lines (PANC1 and BxPC3) using NanoString, a digital technology based on direct multiplexed measurement of nucleic acids through fluorescent barcodes^19^. *NUPR1* was identified as one of the top-five erastin-induced genes in both PANC1 and BxPC3 cells (Fig. 1a, b). Quantitative polymerase chain reaction (qPCR) confirmed that both erastin and RSL3 induced the upregulation of *NUPR1* mRNA in four human PDAC cell lines (PANC1, BxPC3, MiaPaCa2, and CFPAC1), primary human PDAC cells (which we will refer to as “pHsPDAC”), as well as mouse PDAC cell lines (mPDAC) from *Pdx1-Cre;K-Ras*^*G12D/+*^ mice (Supplementary Fig. 1a). Western blot further confirmed the upregulation of NUPR1 protein expression in PANC1, pHsPDAC, and mPDAC cells in response to erastin or RSL3 (Supplementary Fig. 1b). Endoplasmic reticulum (ER) stress is strongly induced in the context of ferroptosis^20^. Notably, the knockdown of activating transcription factor 4 (*ATF4*), a key transcription factor involved in ER stress with anti-ferroptosis activity^21,22^, blocked erastin-induced or RSL3-induced *NUPR1* mRNA expression in PANC1 cells (Supplementary Fig. 1c, d). These findings indicate that ATF4 facilitates the upregulation of NUPR1 in ferroptosis.

To determine whether NUPR1 is a regulator of ferroptosis, we measured cell viability in wild-type (WT) and *Nupr1*^−*/*−^ mPDAC cells. *Nupr1* deletion increased erastin-induced or RSL3-induced growth inhibition (Fig. 1c) and lipid reactive oxygen species (ROS) formation (Fig. 1d) in mPDAC cells, and this effect could be completely reverted by ferroptosis inhibitors (e.g., ferrostatin-1 or liproxstatin-1), but not by inhibitors of apoptosis (e.g., Z-VAD-FMK) or necroptosis (e.g., necrosulfonamide). We confirmed these observations in human *NUPR1*-knockdown PDAC cell lines, including PANC1 and MiaPaCa2 cells (Supplementary Fig. 2). The increased ferroptosis sensitivity was reversed by re-expression of *Nupr1* cDNA in *Nupr1*^−*/*−^ mPDAC cells (Fig. 1d, e). Collectively, these findings demonstrate that NUPR1 is a negative regulator of ferroptosis.

### NUPR1 inhibits iron-dependent oxidative damage in ferroptosis

**Fig. 2:**
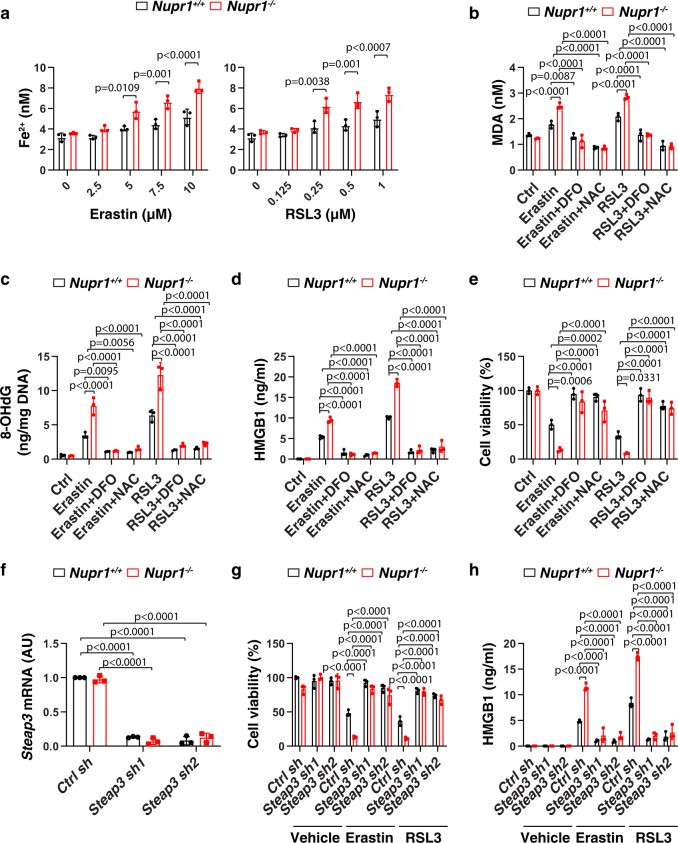
NUPR1 inhibits iron-dependent oxidative damage in ferroptosis. **a** Fe^2+^ levels in indicated mPDAC cells following treatment with erastin or RSL3 for 24 h (*n* = 3 well/group, two-way ANOVA with Tukey’s multiple comparisons test on all pairwise combinations). **b**–**e** Indicated mPDAC cells were treated with erastin (10 µM) or RSL3 (1 µM) in the absence or presence of DFO (100 µM) or NAC (1 mM) for 24 h, and then intracellular MDA (**b**), intracellular 8-OHdG (**c**), extracellular HMGB1 (**d**), and cell viability (**e**) were assayed (*n* = 3 well/group, two-way ANOVA with Tukey’s multiple comparisons test on all pairwise combinations). **f** qPCR analysis of *Steap3* mRNA in indicated mPDAC cells (*n* = 3 well/group, two-way ANOVA with Tukey’s multiple comparisons test on all pairwise combinations). **g**, **h** Indicated mPDAC cells were treated with erastin (10 µM) or RSL3 (1 µM) or vehicle (0.01% dimethyl sulfoxide) for 24 h, and then extracellular HMGB1 (**g**) and cell viability (**h**) were determined (*n* = 3 well/group, two-way ANOVA with Tukey’s multiple comparisons test on all pairwise combinations). Data in **a**–**h** are presented as mean ± SD. The results in **a**–**h** are representative of those from 2 to 3 independent experiments with three technical replicates each.

Iron exists in two oxidation states (ferrous [Fe^2^] or ferric [Fe^3+^]), while Fe^2+^ accumulation is an early signal to initiate ferroptosis^8^. Our biochemical analyses revealed that *Nupr1*^−/−^ mPDAC cells contained higher intracellular Fe^2+^ levels compared to *Nupr1*^*+/+*^ cells in response to erastin or RSL3 (Fig. 2a). The increased oxidative stress caused by iron overload may induce ferroptosis through targeting membrane lipids or DNA^23,24^. Consequently, the depletion of *Nupr1* increased erastin-induced or RSL3-induced lipid peroxidation and oxidative DNA damage in mPDAC cells as measured by quantifying malondialdehyde (MDA) or 8-hydroxy-2-deoxy guanosine (8-OHdG), respectively (Fig. 2b, c). As expected, the release of high-mobility group box 1 (HMGB1), a typical DAMP involved in oxidative stress and cell death response^9^, was increased in *Nupr1*^−*/*−^ mPDAC cells following treatment with erastin or RSL3 (Fig. 2d).

Conversely, the iron chelator deferoxamine (DFO) or the antioxidant N-acetylcysteine (NAC) blocked erastin-induced or RSL3-induced cell death in *Nupr1*^−/−^ mPDAC cells (Fig. 2e), an effect that was associated with decreased production or release of MDA, 8-OHdG, or HMGB1 (Fig. 2b–d). Similarly to *Nupr1*^−*/*−^ mPDAC cells, *NUPR1*-knockdown PANC1 cells exhibited increased MDA, 8-OHdG, and HMGB1 release during ferroptotic cell death, which could be reversed by the addition of DFO or NAC (Supplementary Fig. 3). Of note, the shRNA-mediated knockdown of six-transmembrane epithelial antigen of the prostate 3 (*Steap3*), an enzyme responsible for converting Fe^3+^ to Fe^2+^, blocked erastin-induced or RSL3-induced cell death and HMGB1 release in *Nupr1*^−*/*−^ mPDAC cells (Fig. 2f–h). Together, these findings indicate that NUPR1 blocks ferroptosis through the inhibition of iron-dependent oxidative damage.

### LCN2 acts as an effector gene of NUPR1 in blocking ferroptosis

**Fig. 3:**
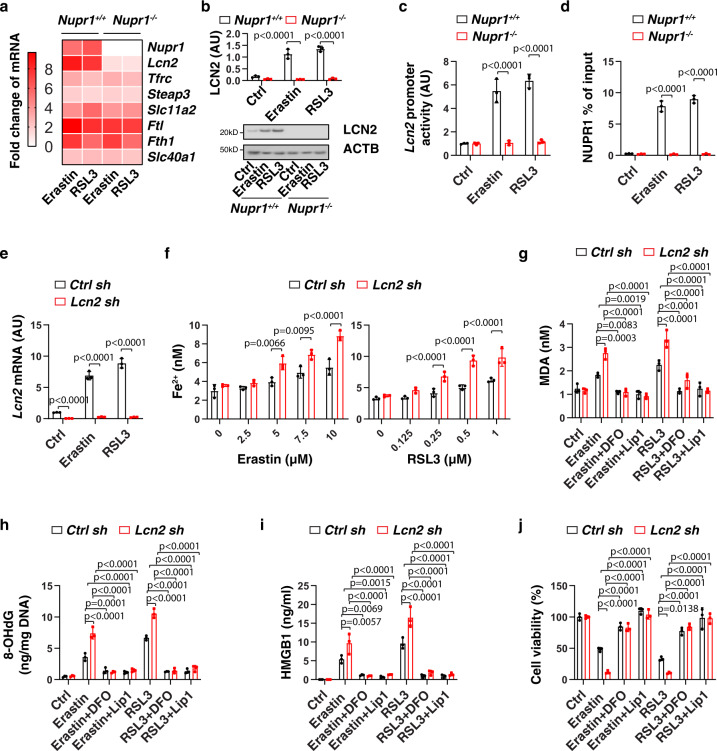
LCN2 acts as an effector gene of NUPR1 in blocking ferroptosis. **a** Heatmap of relative mRNA levels of iron metabolism-associated genes in *Nupr1*^*+/+*^ and *Nupr1*^−*/*−^ mPDAC cells following treatment with erastin (10 µM) or RSL3 (1 µM) for 24 h. **b**, **c** Analysis of LCN2 protein expression and *Lcn2* promoter activity in *Nupr1*^*+/+*^ and *Nupr1*^−*/*−^ mPDAC cells following treatment with erastin (10 µM) or RSL3 (1 µM) for 24 h (*n* = 3 well/group, one-tailed *t* test). **d** Binding of NUPR1 to *Lcn2* promoter was analyzed using ChIP-qPCR in indicated mPDAC cells following treatment with erastin (10 µM) or RSL3 (1 µM) for 24 h (*n* = 3 well/group, one-tailed *t* test). **e** qPCR analysis of *Lcn2* mRNA in indicated mPDAC cells following treatment with erastin (10 µM) or RSL3 (1 µM) for 24 h (*n* = 3 well/group, one-tailed *t* test). **f** Fe^2+^ levels in indicated mPDAC cells following treatment with erastin or RSL3 for 24 h (*n* = 3 well/group, two-way ANOVA with Tukey’s multiple comparisons test on all pairwise combinations). **g**–**j** Indicated mPDAC cells were treated with erastin (10 µM) or RSL3 (1 µM) in the absence or presence of DFO (100 µM) or liproxstatin-1 (1 µM) for 24 h, and then intracellular MDA (**g**), intracellular 8-OHdG (**h**), extracellular HMGB1 (**i**), and cell viability (**j**) were quantified (*n* = 3 well/group, two-way ANOVA with Tukey’s multiple comparisons test on all pairwise combinations). Data in **b**–**j** are presented as mean ± SD. The results in **a**–**j** are representative of those from 2 to 3 independent experiments with three technical replicates each.

The levels of intracellular iron are determined by its uptake, storage, release, and metabolism^25^. In brief, after uptake by transferrin receptor (TFRC, also known as TFR1), Fe^3+^ is reduced to Fe^2+^ by STEAP3 and then released from the endosome to the cytoplasm by solute carrier family 11 member 2 (SLC11A2, also known as DMT1). Ferritin, including ferritin light chain (FTL) and ferritin heavy chain 1 (FTH1), functions as a major iron storage protein. Finally, the release of Fe^2+^ into the extracellular space requires iron transporters, such as solute carrier family 40 member 1 (SLC40A1, also known as ferroportin-1) and yet another ion transporter, LCN2. Among the key genes for iron metabolism (e.g., *Lcn2*, *Tfrc*, *Steap3*, *Slc11a2*, *Ftl*, *Fth1*, and *Slc40a1*), erastin-induced or RSL3-induced the upregulation of *Lcn2* was completely blocked in *Nupr1*^−*/*−^ mPDAC cells (Fig. 3a). Consistent with the mRNA assay, erastin-induced or RSL3-induced protein expression of LCN2 was abolished in *Nupr1*^−*/*−^ mPDAC cells (Fig. 3b). Luciferase reporter gene (Fig. 3c) and chromatin immunoprecipitation (Fig. 3d) assays further revealed that *Lcn2* is a direct target gene of NUPR1 in mPDAC cells during ferroptosis. As expected, the knockdown of *ATF4* by shRNA suppressed *LCN2* mRNA expression in PANC1 cells following erastin or RSL3 treatment (Supplementary Fig. 1e). However, overexpression of ATF4 failed to induce *Lcn2* upregulation in *Nupr1*^−*/*−^ mPDAC cells following erastin or RSL3 treatment (Supplementary Fig. 1f, g). These findings confirm that ATF4-dependent NUPR1 expression is required for subsequent LCN2 expression during ferroptosis.

We next examined whether the genetic silence of LCN2 has a pro-ferroptotic phenotype similar to that of *Nupr1*-deficient cells. Indeed, shRNA-based *Lcn2/LCN2* suppression increased Fe^2+^ accumulation, oxidative damage (MDA and 8-OHdG), HMGB1 release and cell death in mPDAC (Fig. 3e–j) or PANC1 (Supplementary Fig. 4) cells following treatment with erastin or RSL3, which was reversed by DFO or the ferroptosis inhibitor liproxstatin-1. These findings indicate that LCN2 plays a similar role as NUPR1 in the inhibition of ferroptosis.

**Fig. 4:**
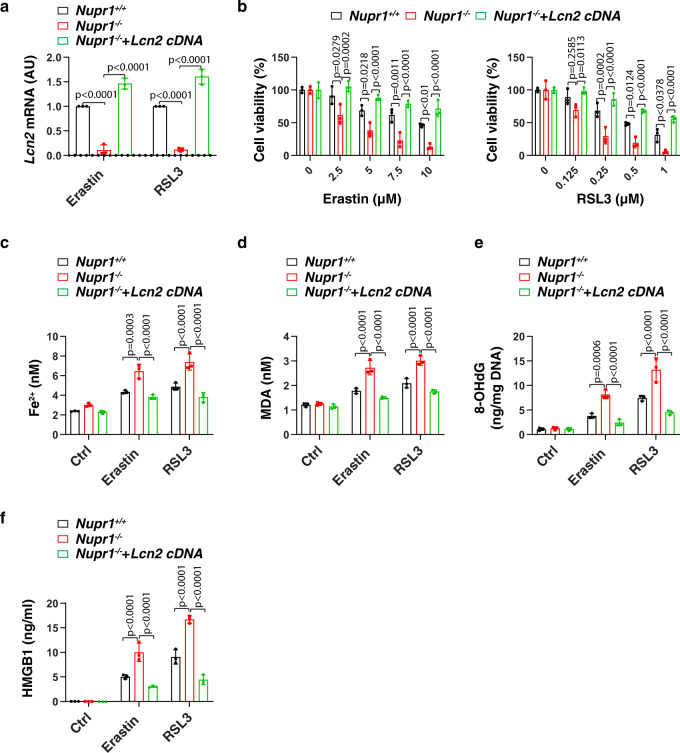
Re-expression of LCN2 rescues ferroptosis resistance in *Nupr1*-deficient cells. **a** qPCR analysis of *Lcn2* mRNA in indicated mPDAC cells following treatment with erastin (10 µM) or RSL3 (1 µM) for 24 h (*n* = 3 well/group, two-way ANOVA with Tukey’s multiple comparisons test on all pairwise combinations). **b** Cell viability in indicated mPDAC cells following treatment with erastin or RSL3 for 24 h (*n* = 3 well/group, two-way ANOVA with Tukey’s multiple comparisons test on all pairwise combinations). **c**–**f** Indicated mPDAC cells were treated with erastin (10 µM) or RSL3 (1 µM) for 24 h, and then intracellular Fe^2+^ (**c**), intracellular MDA (**d**), intracellular 8-OHdG (**e**), and extracellular HMGB1 were assayed (*n* = 3 well/group, two-way ANOVA with Tukey’s multiple comparisons test on all pairwise combinations). Data in **a**–**f** are presented as mean ± SD. The results in **a**–**f** are representative of those from 2 to 3 independent experiments with three technical replicates each.

To determine whether the downregulation of LCN2 is essential for the induction of ferroptosis, we re-expressed *Lcn2* in *Nupr1*^−*/*−^ mPDAC cells by transfecting the *Lnc2* gene (Fig. 4a). The transfection enforced expression of *Lcn2* restored ferroptosis resistance in *Nupr1*^−*/*−^ mPDAC cells, which was associated with decreased Fe^2+^ accumulation, oxidative damage (MDA and 8-OHdG), HMGB1 release, and cell death (Fig. 4b–f). Thus, our findings demonstrated that NUPR1 blocks ferroptotic cell death through inducing the expression of the iron transporter LNC2.

### The NUPR1–LCN2 pathway limits the anticancer activity of IKE in vivo

**Fig. 5:**
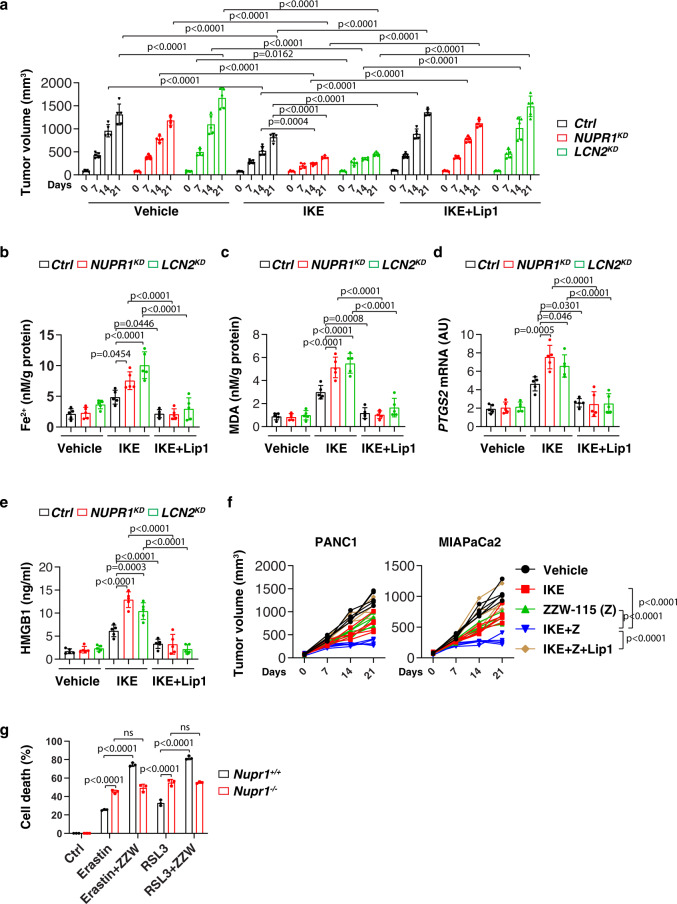
The NUPR1–LCN2 pathway limits ferroptotic cancer cell death in vivo. **a** Athymic nude mice were injected subcutaneously with indicated *PDHA1*-knockdown (*NUPR1*^*KD*^) or *LCN2*-knockdown (*LCN2*^*KD*^) PANC1 cells for 7 days and then treated with IKE (40 mg/kg, i.p., once every other day) in the absence or presence of liproxstatin-1 (10 mg/kg, i.p., once every other day) at day 7 for 2 weeks. Tumor volumes were calculated weekly (*n* = 5 mice/group; two way ANOVA with Tukey’s multiple comparisons test on all pairwise combinations). **b**–**e** In parallel, the levels of Fe^2+^ (**b**), MDA (**c**), or *PTGS2* mRNA (**d**) in isolated tumors and serum HMGB1 (**e**) at day 14 after treatment were assayed (*n* = 5 mice/group; one-way ANOVA with Tukey’s multiple comparisons test on all pairwise combinations). **f** Athymic nude mice were injected subcutaneously with PANC1 or MIAPaCa2 cells for 7 days and then treated with IKE (40 mg/kg, i.p., once every other day) in the absence or presence of ZZW-115 (5 mg/kg, i.p., once every other day) or liproxstatin-1 (10 mg/kg, i.p., once every other day) at day 7 for 2 weeks. Tumor volumes were calculated weekly (*n* = 5 mice/group; two-way ANOVA with Tukey’s multiple comparisons test on all pairwise combinations). **g** Indicated mPDAC cells were treated with erastin (10 µM) or RSL3 (1 µM) in the absence or presence ZZW-115 (2 µM) for 24 h and then cell death was assayed (*n* = 3 well/group, two-way ANOVA with Tukey’s multiple comparisons test on all pairwise combinations). ns: not significant. Data in **a**–**e** and **g** are presented as mean ± SD. The results in **a**–**g** are representative of those from 2 to 3 independent experiments.

We next sought to determine whether the inhibition of the NUPR1–LCN2 pathway can enhance the in vivo anticancer activity of imidazole ketone erastin (IKE), a metabolically stable analog of erastin^26^. Consistent with our in vitro observations, NUPR1-knockdown (*NUPR1*^*KD*^) or LCN2-knockdown (*LCN21*^*KD*^) PANC1 cells were more sensitive to IKE-induced tumor suppression compared to control groups in vivo, in a model in which immunodeficient mice were bearing human PDAC cells (Fig. 5a). The inhibition of the NUPR1–LCN2 pathway conferred therapy sensitivity that was associated with increased intratumoral Fe^2+^ (Fig. 5b) or MDA levels (Fig. 5c), mRNA expression of prostaglandin-endoperoxide synthase 2 (*PTGS2*, an inducible enzyme associated with inflammation and cell death events, including ferroptosis^27^) (Fig. 5d) and circulating HMGB1 protein (Fig. 5e), all of which were inhibited by the ferroptosis inhibitor liproxstatin-1. In contrast, the apoptosis inhibitor Z-VAD-FMK or the necroptosis inhibitor necrosulfonamide had no effects on the increased anticancer activity of IKE in *NUPR1*^*KD*^ or *LCN21*^*KD*^ PANC1 cells (Supplementary Fig. 5). ZZW-115, a potent NUPR1 inhibitor^28^, also increased the anticancer activity of IKE in PANC1 or MIAPaCa2 xenograft mouse models (Fig. 5f). These animal studies support the contention that the NUPR1–LCN2 pathway limits the anticancer activity of IKE. The synergistic effect on cell death by ZZW-115 and erastin or RSL3 was diminished in *Nupr1*^−*/*−^ cells, but not in *Nupr1*^*+/+*^ cells (Fig. 5g), indicating that ZZW-115 bound NUPR1 possesses dominant negative activity or, in other words, the inhibition of NUPR1 is more effective than the loss of NUPR1.

### LCN2 prevents pancreatitis

**Fig. 6:**
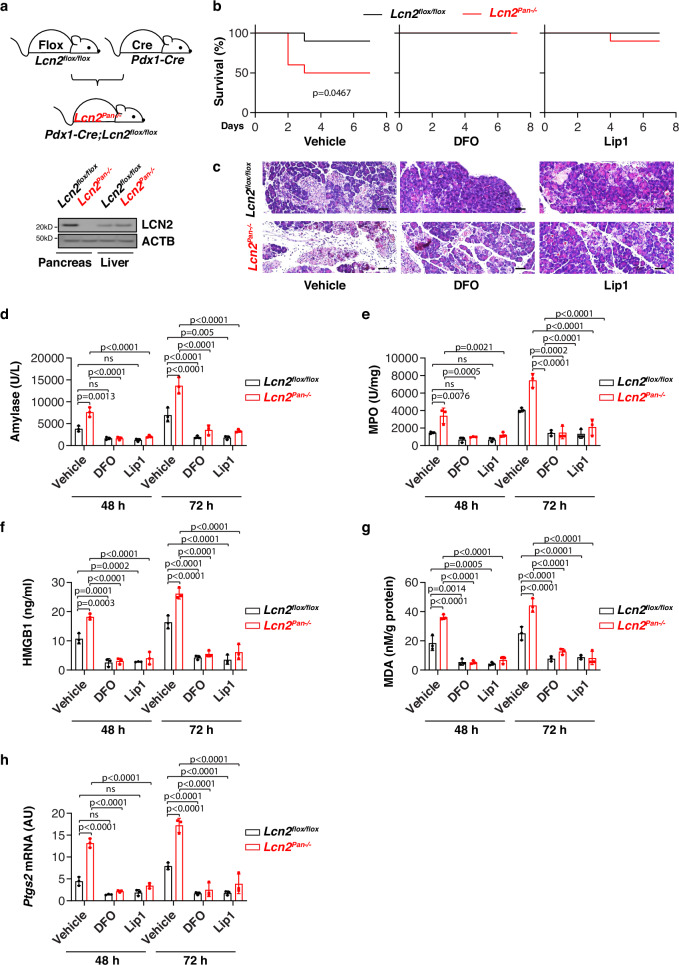
LCN2 prevents pancreatitis through the inhibition of ferroptosis. **a** Western blot analysis of LCN2 expression in the pancreas and liver in *Lcn2*^*flox/flox*^ and pancreas-specific conditional *Lcn2* knockout mice (*Lcn2*^*Pan*−*/*−^). **b** Animal survival in *Lcn2*^*flox/flox*^ and *Lcn2*^*Pan*−*/*−^ mice in response to l-arginine-induced acute severe pancreatitis in the absence or presence of liproxstatin-1 or DFO (*n* = 10 mice/group; one-sided Log-rank [Mantel–Cox] test). **c**–**h** In parallel, hematoxylin and eosin-stained pancreatic sections (**c**), serum amylase (**d**), pancreatic MPO (**e**), serum HMGB1 (**f**), pancreatic MDA (**g**), and mRNA expression of *Ptgs2* in the pancreas (**h**) were assayed at the indicated time points (*n* = 3 mice/group; two-way ANOVA with Tukey’s multiple comparisons test on all pairwise combinations). Bar = 50 μm. ns: not significant. Data in **d**–**h** are presented as mean ± SD. The results in **b**–**h** are representative of those from two independent experiments. The results in (**a**) are from an independent experiment.

Excess iron is stored in multiple organs, including the pancreas, which may cause tissue injury and inflammation under pathologic conditions^29^. We further examined the effects of *Lcn2* depletion-mediated iron accumulation on l-arginine-induced acute pancreatitis in mice, a widely used experimental model that can cause pancreatic oxidative injury, sterile inflammation, and extensive necrosis^30^. We generated pancreas-specific *Lcn2*-knockout mice (*Pdx1-Cre;Lcn2*^*flox/flox*^, termed *Lcn2*^*Pan*−*/*−^ mice) by crossing *Lcn2*^*flox/flox*^ and *Pdx1-Cre* mice. Western blot confirmed the expression of LCN2 was diminished in the pancreas (but not in the liver) from *Lcn2*^*Pan*−*/*−^ mice, but not from control *Lcn2*^*flox/flox*^ mice (Fig. 6a). *Lcn2*^*Pan*−*/*−^ mice were more sensitive to l-arginine-induced acute pancreatitis, hence exhibiting increased mortality (Fig. 6b), aggravated pancreatic histological damage (Fig. 6c), and elevated serum amylase (a diagnostic biomarker of acute pancreatitis) (Fig. 6d), pancreatic myeloperoxidase (MPO, a marker of neutrophil recruitment) (Fig. 6e), serum HMGB1 (Fig. 6f), pancreatic MDA (Fig. 6g), and *Ptgs2* mRNA (Fig. 6h). This phenotype of *Lcn2* depletion-mediated acute pancreatitis could be prevented by treatment with the ferroptosis inhibitor liproxstatin-1 or the iron chelator DFO (Fig. 6b–h). Collectively, these studies suggest that LCN2 exerts a protective effect on acute pancreatitis potentially through the inhibition of ferroptotic response.

### Prognostic significance of the NUPR1–LCN2 pathway in human PDAC

**Fig. 7:**
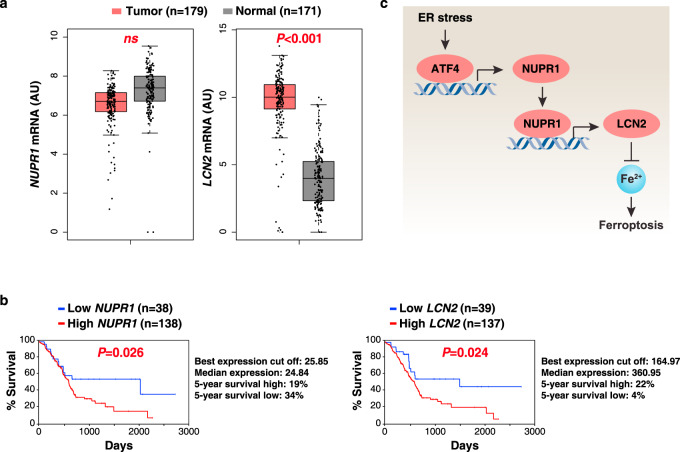
Prognostic significance of the NUPR1–LCN2 pathway in human PDAC. **a** Upregulation of *LCN2* (but not *NUPR1*) gene expression within the tumor from PDAC patients compared to normal controls using datasets from the Cancer Genome Atlas (TCGA) (one-tailed *t* test). The data are presented as box-and-whisker plots. Boxes represent the median and the 25th and 75th percentiles. ns: not significant. *n* is number of cases. **b** Kaplan–Meier survival analysis of *NUPR1* and *LCN2* gene expression in PDAC patients using TCGA datasets (one-sided Log-rank [Mantel–Cox] test). *n* is number of cases. **c** Schematic representation of the role of NUPR1 in the regulation of ferroptosis in human PDAC cells.

Previous animal studies indicate that NUPR1 and LCN2 may play an oncogene-like role in PDAC^31,32^. To test this possibility, we carried out bioinformatics analyses using a publicly available gene expression dataset: the Cancer Genome Atlas (TCGA). *LCN2* (but not *NUPR1*) mRNA expression was upregulated in the PDAC tumor group compared to the normal group (Fig. 7a). An overall survival assay revealed that a low expression of *NUPR1* or *LCN2* was correlated with increased survival of PDAC patients (Fig. 7b). These analyses indicate that activation of the NUPR1–LCN2 pathway may contribute to the development of human PDAC.

## Discussion

There is a clear role for transcription factors in human health and disease, highlighting the importance of continued efforts for understanding gene expression modulation in physiological and pathological processes, including cell death^33^. In this study, we demonstrated that NUPR1 is critical for activating a transcriptional program that may block ferroptotic cell death or tissue injury through the upregulation of LCN2 expression (Fig. 7c), providing a potential strategy for the treatment of ferroptosis-related diseases.

NUPR1 is a member of AT hook-containing chromosomal DNA-binding proteins that was first identified and cloned in a study of pancreatitis-induced tissue injury^34^. Like other architectural chromatin-binding proteins, NUPR1 participates in a wide range of DNA-relevant events, such as gene transcription, DNA repair, and chromosome recombination^35–39^. NUPR1 is also a multifunctional stress-inducible protein that is produced in response to diverse environmental stresses, including oxidative damage and the unfolded protein response^40–45^. ATF4, a repressor of ferroptosis in PDAC cells^22^, has been shown to regulate NUPR1 expression in response to various stresses, including ER stress^46^. We found that ATF4 is required for NUPR1 expression in response to ferroptosis activators. NUPR1 regulates cellular processes mainly by activating its target genes^40,47^. Our current study establishes an inhibitory role for NUPR1 in regulating ferroptosis through directly upregulating LCN2 expression. We found that the depletion of NUPR1 or LCN2 had a similar pro-ferroptotic phenotype in human or mouse PDACs, whereas re-expression of LCN2 reversed the exaggerated ferroptosis observed in *Nupr1*^−*/*−^ cells. Combined with other studies on models of apoptosis^31,48^, necroptosis^28,44^ and autophagy-dependent cell death^41,49,50^, the present study suggests that the activation of NUPR1 may play a universal cytoprotective role. Since ATF4 has been shown to promote the expression of solute carrier family 7 member 11 (SLC7A11, a component of the antiporter system xc^−^ that inhibits ferroptosis by importing the amino acid cystine for glutathione production in cells)^21^, it is necessary to investigate whether NUPR1 is also involved in ATF4-mediated SLC7A11 expression in the future.

Our studies reveal a role for NUPR1 in preventing intracellular iron accumulation in response to ferroptosis activators. Ferroptosis is generally recognized as an iron-dependent oxidative death, which can be suppressed by iron chelators, the depletion of the iron uptake receptor TFRC or the overexpression of the iron storage protein ferritin^8^. Given that depletion of NUPR1 increased iron accumulation induced by classical ferroptosis activators (erastin or RSL3), the expression levels of core iron metabolism-associated genes were measured in WT and *Nupr1*^−*/*−^ cells. Such analyses led to the conclusion that *Lcn2*, but none of the other iron metabolism-relevant genes, functions as a direct downstream target gene regulated by NUPR1. Although the function of LCN2 in iron metabolism is still unclear, it has been suggested to be involved in the extrusion of iron from the intracellular to the extracellular compartment^51–53^. Blocking NUPR1-dependent LCN2 expression significantly increased intracellular iron concentrations and subsequent oxidative damage, including lipid peroxidation and DNA damage. These observations established a NUPR1-dependent LCN2-regulatory pathway for ferroptosis (Fig. 7c).

While the function of mitochondria in ferroptosis is context-dependent^3,54,55^, mitochondria are widely recognized as a major source of iron-induced ROS^56^. Mitochondria also utilize iron for the synthesis of heme and iron sulfur, which can form a feedback to amplify or diminish ferroptosis^57,58^. In view of previously established links between the downregulation of NUPR1 expression and the increased mitochondria-dependent apoptosis in some types of cells^31,48^, it is possible that NUPR1 downregulation-mediated iron accumulation functions as a common upstream signal to trigger mitochondrial dysfunction, thus compromising cellular survival and fitness by negatively affecting aerobic glycolysis, oxidative phosphorylation, and fatty acid synthesis^44,54,59–62^.

Our findings highlight a potential functional role of the NUPR1–LCN2 pathway in the regulation of ferroptosis-related tumor therapy. While the deletion of *Nupr1* in mice prevents mutated *Kras*-induced tumorigenesis^31,63^, we found that the pharmacological or genetic inhibition of the NUPR1–LCN2 pathway enhanced anticancer activity of ferroptosis activators in PDAC cells in vitro or in preclinical mouse models. Ferroptosis seems to play a dual role in tumor biology, which is context-dependent. On the one hand, ferroptosis activators can kill cancer cells or active antitumor immunity to suppress tumor growth^64–66^. On the other hand, ferroptosis-mediated inflammatory response may promote tumor growth through the release of DAMPs^67,68^. Further exploration of the cellular and functional relevance of ferroptosis in the tumor microenvironment would be important to develop better anticancer therapeutic strategies.

We addressed a potential pathologic link between ferroptosis and pancreatitis using *Lcn2*^*Pan*−*/*−^ mice, knowing that excessive ferroptosis can compromise the function of entire organs including the kidney, liver, brain, heart, and pancreas^7^. Compared to WT mice, *Lcn2*^*Pan*−*/*−^ mice exhibited increased sensitivity to experimental pancreatitis-mediated animal death. In contrast, the inhibition of ferroptosis by liproxstatin-1 or by using the iron chelator DFO protected against pancreatic injury, lipid peroxidation, HMGB1 release, and PTGS2 expression, leading to prolonged animal survival in *Lcn2*^*Pan*−*/*−^ mice. In addition to PTGS2, transferrin receptor (TFRC) and acyl-CoA synthetase long chain family member 4 (ACSL4) have also been used to monitor ferroptotic response under different conditions^59,69^. However, the specific quantitative markers of ferroptotic death in vivo have not yet been determined. Given that LCN2 plays a wide role in preventing infection and sterile inflammation in various animal models^51–53^, LCN2-mediated ferroptosis resistance may be a common mechanism driving cell protection and reducing morbidity and mortality, a possibility that awaits further investigation in future studies.

In summary, our studies reveal a potential negative role for NUPR1 in ferroptosis. The crosstalk between gene transcription and iron metabolism may have broad implications for modulating ferroptosis in pathologic conditions.

## Methods

### Reagents

Erastin (S7242), ferrostatin-1 (S7243), liproxstatin-1 (S7699), RSL3 (S8155), IKE (S8877), DFO (S5742), NAC (S5804), Z-VAD-FMK (S7023), and necrosulfonamide (S8251) were purchased from Selleck Chemicals. l-arginine (A5131) was purchased from Sigma-Aldrich. ZZW-115 (PC-36152) was purchased from ProbeChem. The antibody to ACTB (3700; RRID:AB_2242334; 1:1000) was obtained from Cell Signaling Technology. The antibody to LCN2 (ab63929; RRID:AB_1140965; 1:500) was obtained from Abcam. The antibody to NUPR1 (SAB2109172 [RRID:AB_2868575; 1:500] or sc-23283 [RRID:AB_2157971; 1:100]) was obtained from Sigma-Aldrich or Santa Cruz Biotechnology.

### Cell culture

The PANC1 (CRL-1469) and MIAPaCa2 (CRL-1420) cell lines were obtained from the American Type Culture Collection. PHsPDAC cells were a gift from Yangchun Xie and they were generated from patient tumor specimens^70,71^. WT and *Nupr1*^−/−^ mPDAC cells were generated from *Nupr1*^*+/+*^*;Pdx1-cre;LSL-Kras*^*G12D*^ or *Nupr1*^*−/−*^*;Pdx1-cre;LSL-Kras*^*G12D*^ mice, respectively, which was a gift from Juan Iovanna (Centre de Recherche en Cancérologie de Marseille, INSERM, France). WT and *Nupr1*^−/−^ mPDAC were used at <10 passages. These cells were cultured in Dulbecco’s modified Eagle’s medium (Thermo Fisher Scientific, 11995073) supplemented with 10% heat-inactivated fetal bovine serum (Thermo Fisher Scientific, A3840001) and 1% penicillin and streptomycin (Thermo Fisher Scientific, 15070-063) at 37 °C, 95% humidity, and 5% CO_2_. Cell line identity was validated by short tandem repeat profiling, and routine mycoplasma testing was negative for contamination.

### Cell viability assay

Cell viability was assayed by a CCK8 kit (Dojindo Laboratories, CK04). In brief, cells were seeded into 96-well plates and incubated with the indicated treatments. Subsequently, 100 μl of fresh medium was added to cells containing 10 μl of CCK-8 solutions and incubated for 2 h (37°C, 5% CO_2_). Absorbance at 450 nm was measured using a microplate reader (Cytation 5 Cell Imaging Multi-Mode Reader). In addition, a Countess II FL Automated Cell Counter (Thermo Fisher Scientific) was used to assay the percentages of dead cells after cell staining with 0.4% trypan blue solution (Thermo Fisher Scientific, T10282).

### RNAi and gene transfection

Mouse *Nupr1*-cDNA (EX-Mm07534-M02) or *Lcn2*-cDNA (EX-Mm03601-M02) or or *Atf4*-cDNA (EX-Mm20335-M02) were purchased from GeneCopoeia. Human *NUPR1*-shRNA (sequence: CCGGGGATGAATCTGACCTCTATAGCTCGAGCTATAGAGGTCAGATTCATCCTTTTTG), human *LCN2*-shRNA (sequence: CCGGGCTGGGCAACATTAAGAGTTACTCGAGTAACTCTTAATGTTGCCCAGCTTTTTG), human *ATF4*-shRNA-1 (sequence: CCGGGCCTAGGTCTCTTAGATGATTCTCGAGAATCATCTAAGAGACCTAGGCTTTTT), human *ATF4*-shRNA-2 (sequence: CCGGGTTGGTCAGTCCCTCCAACAACTCGAGTTGTTGGAGGGACTGACCAACTTTTT), mouse *Lcn2*-shRNA (sequence: CCGGCCAGGACTCAACTCAGAACTTCTCGAGAAGTTCTGAGTTGAGTCCTGGTTTTTG), mouse *Steap3*-shRNA-1 (sequence: CCGGACAGCGAGGTGATGATATATGCTCGAGCATATATCATCACCTCGCTGTTTTTTG), and mouse *Steap3*-shRNA-2 (sequence: CCGGCGCTCCCGTCCATTGCTAATTCTCGAGAATTAGCAATGGACGGGAGCGTTTTTG) were obtained from Sigma-Aldrich. Transfection with shRNA or cDNA was performed with Lipofectamine 3000 (Invitrogen, L3000-015) according to the manufacturer’s instructions.

### NanoString nCounter analysis

Total RNA from tumors was extracted using the RNeasy Plus Mini Kit (QIAGEN, 74136) and hybridized with the NanoString nCounter Human PanCancer Pathways Panel Code Set (NanoString) with 770 genes from 13 cancer-associated canonical pathways, including MAPK, STAT, PI3K, RAS, cell cycle, apoptosis, Hedgehog, Wnt, DNA damage control, transcriptional regulation, chromatin modification, and TGF-β^19^. Briefly, 100 ng of scaled RNA was hybridized with biotin-labeled capture probes and fluorescently labeled reporter probes for 18 h at 65 °C. Subsequently, the strip tubes were placed into the nCounter Prep Station for automated sample purification and subsequent reporter capture. Each sample was scanned for 600 FOV on the nCounter Digital Analyzer. NanoString results were produced from RCC files using nSolver Analysis Software 3.0.

### qPCR analysis

Total RNA was extracted and purified from cultured cells or tissues using the RNeasy Plus Mini Kit (QIAGEN, 74136) according to the manufacturer’s instructions. The RNA was quantified by determining absorbance at 260 nm. One microgram of total RNA from each sample was reverse-transcribed into cDNA using the iScript cDNA synthesis kit (Bio-Rad, 170-8891) in a volume of 20 μl; cDNA from cell samples was amplified. The qPCR was performed using SsoFast EvaGreen Supermix (Bio-Rad, 172-5204) on the C1000 Touch Thermocycler CFX96 Real-Time System (Bio-Rad) according to the manufacturer’s protocol. Analysis was performed using Bio-Rad CFX Manager software 3.1 (Bio-Rad). The specific primers were listed in Supplementary Table 1. The gene expression was calculated via the 2^−ΔΔCt^ method and normalized to *18SRNA/18srna*^72^. The relative concentrations of mRNA were expressed in arbitrary units based on the untreated group, which was assigned a value of 1.

### Western blot

Cells were lysed in 1× cell lysis buffer (Cell Signaling Technology, 9803) containing protease inhibitor (ROCHE, 11836153001) on ice for 10 min. After centrifugation at 14,000 × *g* for 15 min at 4 °C, the supernatants were collected and quantified using BCA assay (Thermo Fisher Scientific, 23225). The 30 μg of each sample was resolved on 4–12% Criterion XT Bis–Tris gels (Bio-Rad, 3450124) in XT MES running buffer (Bio-Rad, 1610789) and transferred to PVDF membranes (Bio-Rad, 1620233) using the Trans-Blot Turbo Transfer Pack and System (Bio-Rad)^73^. After blocking by TBST containing 5% skim milk for 1 h, the membrane was incubated overnight at 4 °C with various primary antibodies (1:200–1:1000). After incubation with peroxidase-conjugated secondary antibodies (goat anti-rabbit IgG secondary antibody [Cell Signaling Technology, 7074, RRID:AB_2099233, 1:1000]; horse anti-mouse IgG secondary antibody [Cell Signaling Technology, 7076, RRID:AB_330924, 1:1000]; rabbit anti-goat IgG secondary antibody [Abcam, ab6741, RRID:AB_955424, 1:1000]) for 1 h at room temperature, the signals were visualized using enhanced chemiluminescence (Thermo Fisher Scientific, 34095). We collected protein from each cell line in three biologically independent samples and mixed them together for western blot analysis, and repeated twice. The relative intensities of the bands of western blots from three regions were automatically analyzed and normalized to a loading control using the ChemiDoc Touch Imaging System Version 1.2 (Bio-Rad). Source data of images of western blot bands are provided as a Source Data file.

### Biochemical assay

Commercially available enzyme-linked immunosorbant assay (ELISA) kits were used to measure the concentrations or activity of HMGB1 (Shino Test Corporation, ST51011), amylase (BioVision, K711), MPO (BioVision, K744), iron (Abcam, ab83366), MDA (Abcam, ab118970), and 8-OHdG (Cell Biolabs, STA-320) in indicated samples according to the manufacturer’s instructions. Data were normalized to protein or DNA concentration. In addition, C11-BODIPY probe (Thermo Fisher Scientific, D3861) was used to detect lipid ROS in cells.

### Secrete-pair luminescence and ChIP assay

Dual-reporter promoter clones or controls were transfected into two cell lines in duplicates. Indicated WT and *Nupr1*^−*/*−^ cells were transfected with pEZX-PG04-*Lcn*2 promoter Gaussia luciferase/secreted alkaline phosphatase (GeneCopoeia, MPRM39850-PG04). After 48 h, these cells were treated with erastin (10 µM) or RSL3 (1 µM) at indicated times. The *Lcn2* promoter luciferase activity was measured with a Secrete-Pair Dual Luminescence Assay Kit (GeneCopoeia, SPDA-D010) in accordance with the manufacturer’s guidelines. The chromatin immunoprecipitation (ChIP) assay was performed using the Pierce Magnetic ChIP Kit (Thermo Scientific, 26157). This kit contained reagents to lyse cells and extract and solubilize the crosslinked complexes. The complexes were then incubated with anti-NUPR1 antibody (Santa Cruz Biotechnology, sc-23283) and isolated using Pierce Protein A/G Magnetic Beads. After reversing crosslinks and digesting protein, the resulting DNA fragments were purified. One-twentieth of the immunoprecipitated DNA was used in qPCR. The results were shown as a percentage of input.

### Animal model

We conducted all animal care and experiments in accordance with the Association for Assessment and Accreditation of Laboratory Animal Care guidelines and with approval from our Institutional Animal Care and Use Committee (Guangzhou Medical University [#2019075] and UT Southwestern Medical Center [#102605]). All mice were housed under a 12-h light–dark diurnal cycle with controlled temperature (20–25 °C) and relative humidity (40–60%). Food and water were available ad libitum. Experiments were carried out under pathogen-free conditions and the health status of mouse lines was routinely checked by veterinary staff. No wild animals were used in the study. Experiments were carried out with randomly chosen littermates of the same sex and matched by age and body weight. Animals were sacrificed at the indicated time by CO_2_ asphyxia, and blood samples and tissue were collected.

To generate murine subcutaneous tumors, 5 **×** 10^6^ PANC1 or MIAPaCa2 cells in 100 μl PBS was injected subcutaneously to the right of the dorsal midline in 6–8-week-old athymic nude female mice. Once the tumors reached around 80 mm^3^ at day 7, mice were randomly allocated into groups and then treated with IKE (40 mg/kg, i.p., once every other day) in the absence or presence of Z-VAD-FMK (10 mg/kg, i.p., once every other day) or necrosulfonamide (10 mg/kg, i.p., once every other day) or liproxstatin-1 (10 mg/kg, i.p., once every other day) or ZZW-115 or liproxstatin-1 (5 mg/kg, i.p., once every other day) starting at day 7 for 2 weeks. Tumors were measured twice weekly and volumes were calculated using the formula length × width^2^ × *π*/6.

Pancreatic-specific *Lcn2*-knockout mice were generated by crossing floxed *Lcn2* (*Lcn2*^*flox/flox*^) and *Pdx1-Cre* transgenic mice. *Lcn2*^*flox/flox*^ mice were a gift from Bin Gao (National Institutes of Health, USA). *Pdx1-Cre* mice (014647) were purchased from the Jackson Laboratory. All mice were C57BL/6 background. For l-arginine-induced pancreatitis, a sterile solution of l-arginine monohydrochloride (8%) was prepared in normal saline and the pH was adjusted to 7.0. Mice received two hourly intraperitoneal (i.p.) injections of l-arginine (4 g/kg), while controls were administered saline i.p.^74^. In addition, pretreatment with liproxstatin-1 (10 mg/kg) or DFO (100 mg/kg) for 1 h was used in pancreatitis models.

### Bioinformatics analysis

GEPIA (http://gepia.cancer-pku.cn/index.html)^75^, an interactive web server for analyzing the TCGA data, was used to separate the TCGA cohorts into groups with high/low expression of selected genes, which were then used for the prognostic signature validation based on the best cut-off values. The best expression cut-off refers the fragments per kilobase of exon model per million reads mapped (FPKM) value that yields maximal difference with regard to survival between the two groups at the lowest log-rank *P*-value.

### Statistical analysis

Data are presented as mean ± SD except where otherwise indicated. GraphPad Prism 8.4.3 was used to collect and analyze data. Unpaired Student’s *t* tests were used to compare the means of two groups. A one-way (for one independent variable) or two-way (for two independent variables) analysis of variance (ANOVA) with Tukey’s multiple comparisons test was used for comparison among the different groups on all pairwise combinations. Log-rank test was used to compare differences in mortality rates between groups. A two-tailed *P* value of <0.05 was considered statistically significant. The exact value of *n* within the figures and replicates is indicated in the figure legends. We did not exclude samples or animals. No statistical methods were used to predetermine sample sizes, but our sample sizes are similar to those generally employed in the field^26,76^.

### Reporting summary

Further information on research design is available in the Nature Research Reporting Summary linked to this article.

## Supplementary information

Supplementary Information

Reporting Summary

## Source data

Source Data

## Data Availability

All the other data supporting the findings of this study are available within the article and its supplementary information files and from the corresponding author upon reasonable request. GEPIA (http://gepia.cancer-pku.cn/index.html)^75^ was used to analyze the TCGA data of NURP1 (Ensembl ID: ENSG00000176046 [https://www.ncbi.nlm.nih.gov/gene/26471]) or LCN2 (Ensembl ID: ENSG00000148346 [https://www.ncbi.nlm.nih.gov/gene/3934]) gene. Source data are provided with this paper.
