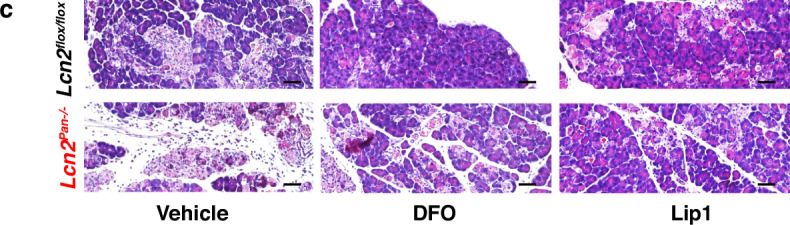# Author Correction: NUPR1 is a critical repressor of ferroptosis

**DOI:** 10.1038/s41467-025-61573-9

**Published:** 2025-07-04

**Authors:** Jiao Liu, Xinxin Song, Feimei Kuang, Qiuhong Zhang, Yangchun Xie, Rui Kang, Guido Kroemer, Daolin Tang

**Affiliations:** 1 The Third Affiliated Hospital, Key Laboratory of Protein Modification and Degradation, Guangzhou Medical University, 510600 Guangdong, China; 2 Department of Surgery, UT Southwestern Medical Center, Dallas, TX 75390 USA; 3 Department of Surgery, University of Pittsburgh, Pittsburgh, PA 15219 USA; 4 Department of Oncology, The Second Xiangya Hospital, Central South University, Changsha, Hunan China; 5 Université Paris Descartes, Sorbonne Paris Cité, 75006 Paris, France; 6 Equipe 11 labellisée Ligue Nationale contre le Cancer, Centre de Recherche des Cordeliers, 75006 Paris, France; 7 Institut National de la Santé et de la Recherche Médicale, U1138 Paris, France; 8 Université Pierre et Marie Curie, 75006 Paris, France; 9 Metabolomics and Cell Biology Platforms, Gustave Roussy Cancer Campus, 94800 Villejuif, France; 10 Pôle de Biologie, Hôpital Européen Georges Pompidou, AP-HP, 75015 Paris, France; 11 Department of Women’s and Children’s Health, Karolinska University Hospital, 17176 Stockholm, Sweden

Correction to: *Nature Communications* 10.1038/s41467-021-20904-2, published online 28 January 2021

In the version of the article initially published, the “DFO, *Lcn2*^*flox/flox*^” image was incorrect and has now been amended in the HTML and PDF versions of the article, as seen in Fig. 6c below.

Original Fig. 6c
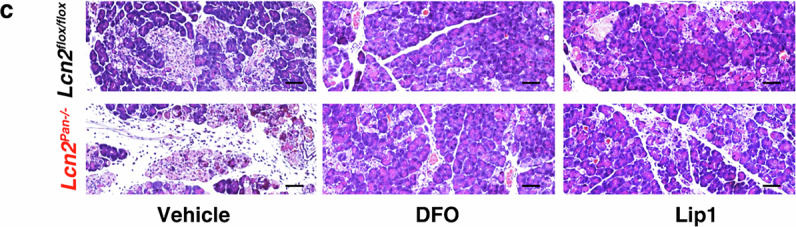


Corrected Fig. 6c