# Supplementary material for: NUPR1 is a critical repressor of ferroptosis
Source: Nat Commun. 2021 Jan 28;12:647. doi: 10.1038/s41467-021-20904-2 (PMC7843652; doi:10.1038/s41467-021-20904-2)
Supplement: Supplementary file 1 — Supplementary Information [file 41467_2021_20904_MOESM1_ESM.pdf]

Supplementary Information

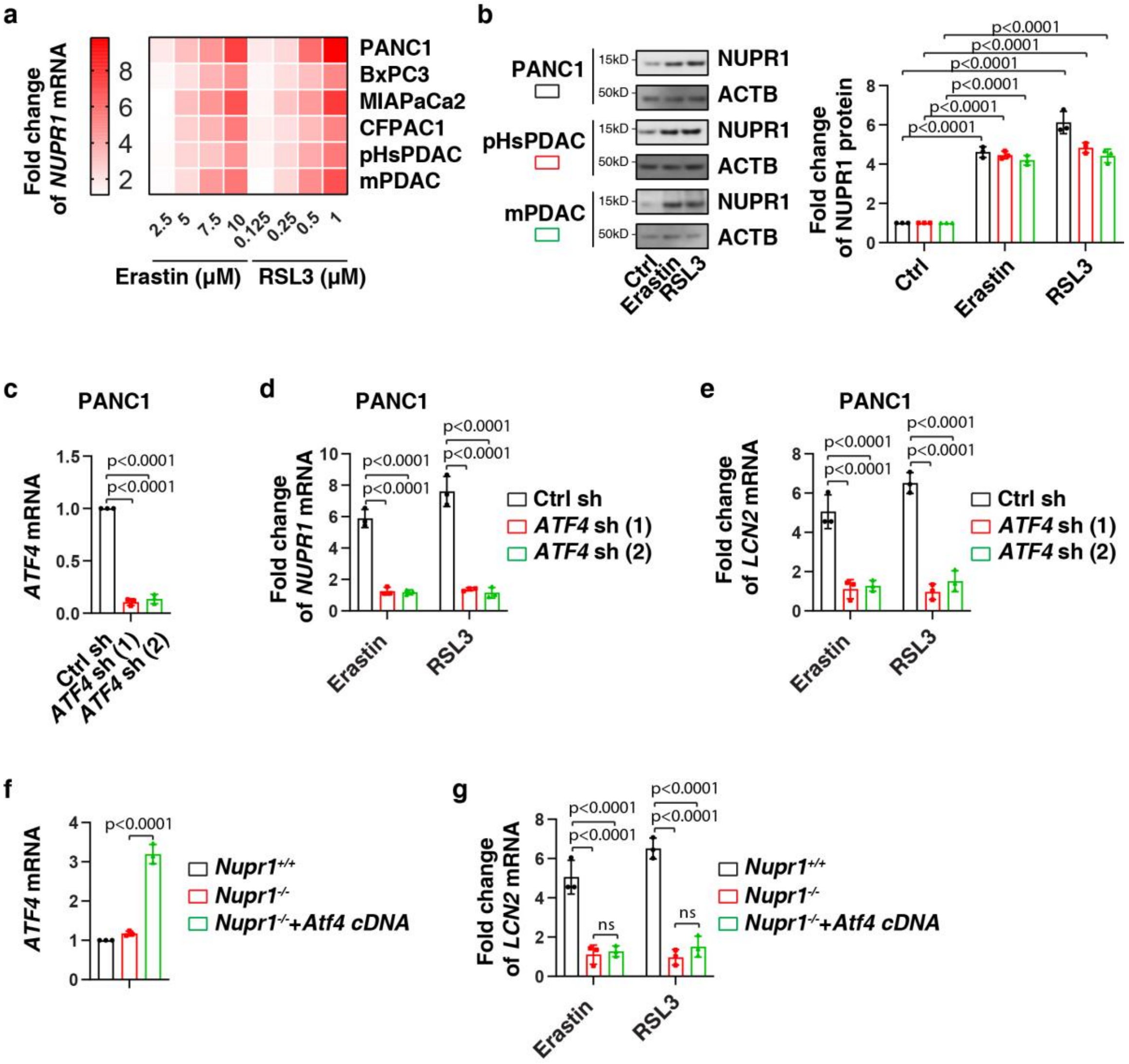

Supplementary Figure 1. ATF4-mediated NUPR1 upregulation in ferroptosis. (a) Heatmap of relative mRNA levels of *NUPR1* in indicated PDAC cells following treatment with erastin or RSL3 for 24 h. (b) Western blot analysis of NUPR1 expression in indicated PDAC cells following treatment with erastin (10 μM) or RSL3 (1 μM) for 24 h (n= 3 well/group, two way ANOVA with Tukey's multiple comparisons test on all pairwise combinations). (c-e) Knockdown of ATF4 by shRNA inhibited NUPR1 or

LCN2 upregulation in PANC1 cells following treatment with erastin (10 μM) or RSL3 (1 μM) for 24 h (n= 3 well/group, one or two way ANOVA with Tukey's multiple comparisons test on all pairwise combinations). (f, g) overexpression of ATF4 failed to induce *Lcn2* upregulation in *Nupr1*$^{-/-}$ mPDAC cells following erastin (10 μM) or RSL3 (1 μM) for 24 h (n= 3 well/group, one or two way ANOVA with Tukey's multiple comparisons test on all pairwise combinations). Data in (b-g) are presented as mean ± SD. The results in (b-g) are representative of those from 2-3 independent experiments with 3 technical replicates each.

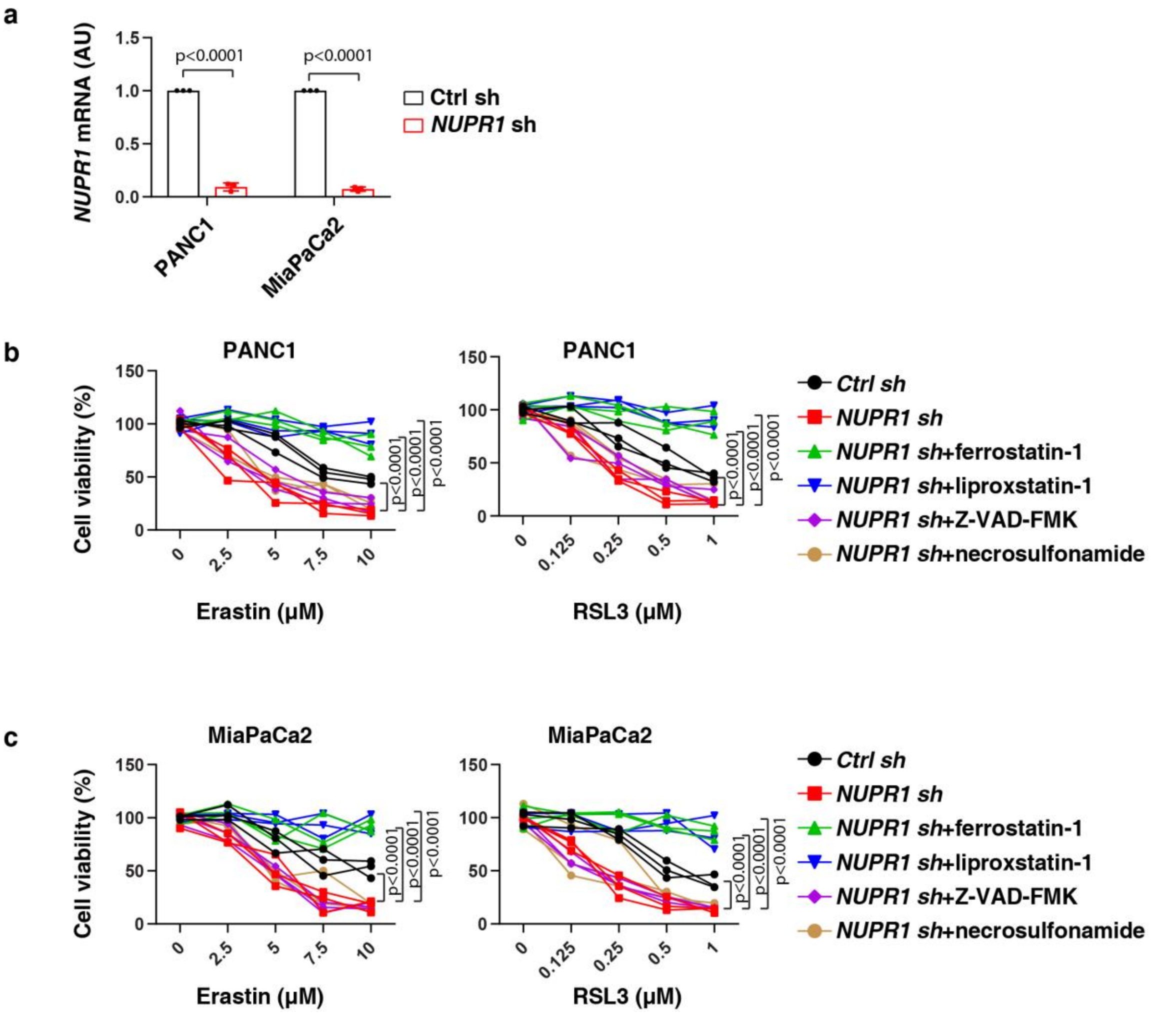

Supplementary Figure 2. NUPR1 acts as a repressor of ferroptosis. (a) Western blot analysis of NUPR1 expression in indicated PANC1 or MiaPaCa2 cells (n= 3 well/group, one-tailed *t* test). (b, c) Indicated PANC1 or MiaPaCa2 cells were treated with erastin or RSL3 in the absence or presence of ferrostatin-1 (1 μM), liproxstatin-1 (1 μM), Z-VAD-FMK (10 μM), or necrosulfonamide (1 μM) for 24 h, and then cell viability was assayed (n= 3 well/group, two way ANOVA with Tukey's multiple comparisons test on all pairwise combinations). Data in (a-c) are presented as mean ± SD. The results in (a-c) are representative of those from 2 independent experiments with 3 technical replicates each.

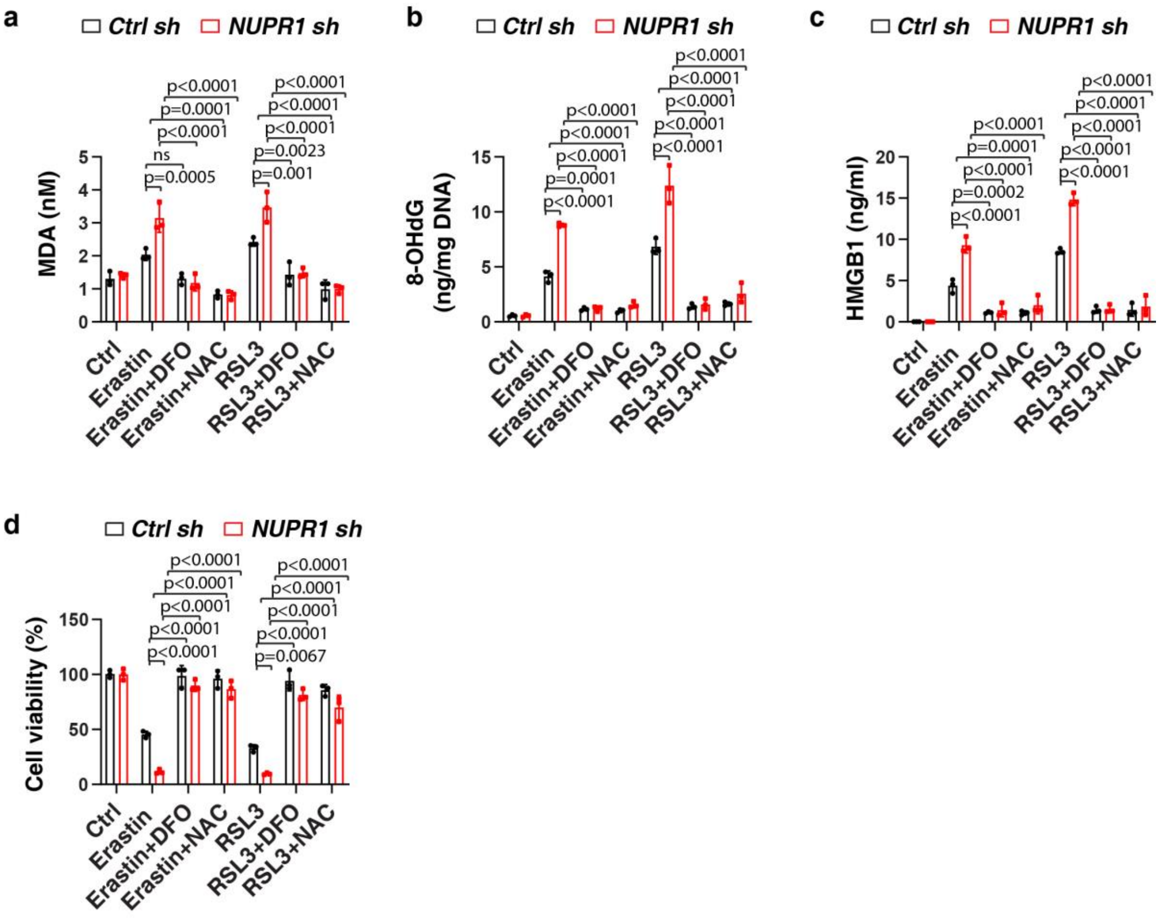

Supplementary **Figure 3. NUPR1 inhibits iron-dependent oxidative damage in ferroptosis.** Indicated PANC1 cells were treated with erastin (10 μM) or RSL3 (1 μM) in the absence or presence of DFO (100 μM) or NAC (1 mM) for 24 h, and then intracellular MDA (a), intracellular 8-OHdG (b), extracellular HMGB1 (c), and cell viability (d) were assayed (n= 3 well/group, two way ANOVA with Tukey's multiple comparisons test on all pairwise combinations). ns: not significant. Data in (a-d) are presented as mean ± SD. The results in (a-d) are representative of those from 2 independent experiments with 3 technical replicates each.

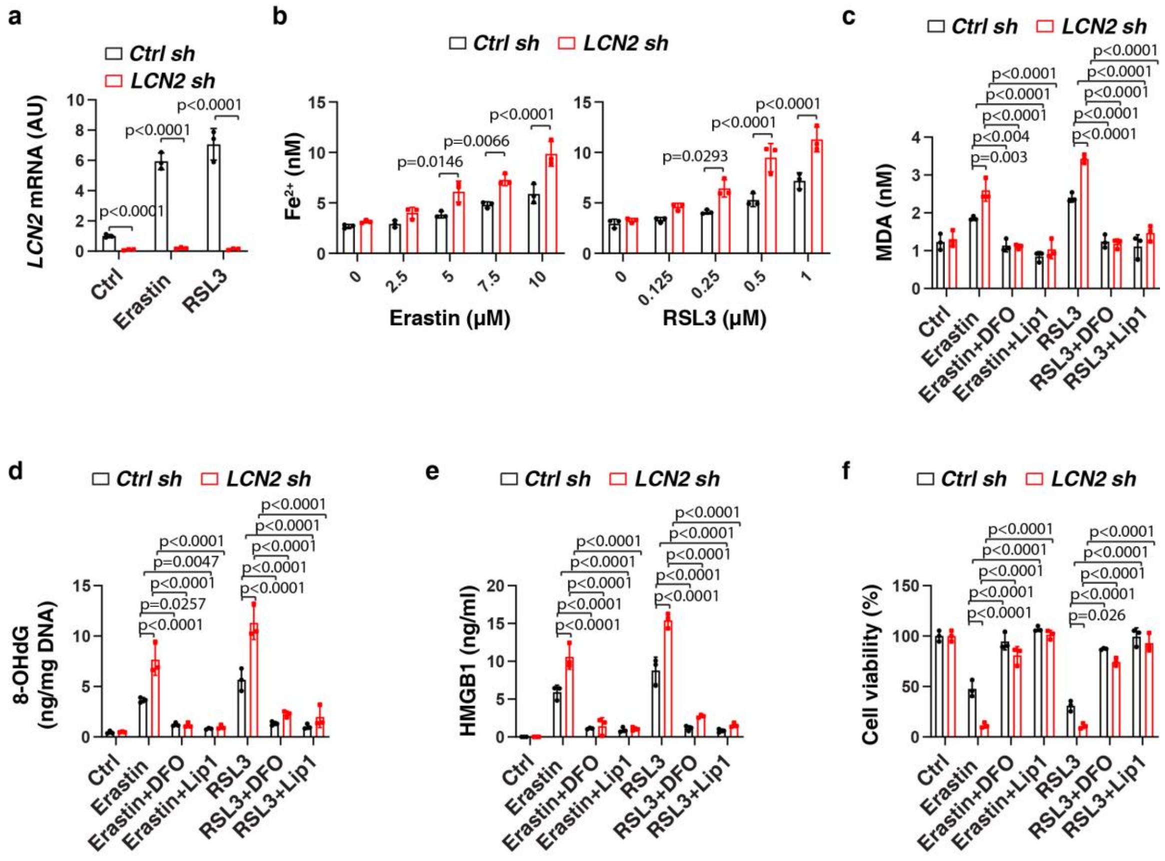

Supplementary Figure 4. LCN2 inhibits ferroptosis. (a) qPCR analysis of *LCN2* mRNA in indicated PANC1 cells following treatment with erastin (10 μM) or RSL3 (1 μM) for 24 h (n= 3 well/group; one-tailed *t* test). (b) Fe^{2+} levels in indicated PANC1 cells following treatment with erastin or RSL3 for 24 h (n= 3 well/group, two way ANOVA with Tukey's multiple comparisons test on all pairwise combinations). (c-f) Indicated PANC1 cells were treated with erastin (10 μM) or RSL3 (1 μM) in the absence or presence of DFO (100 μM) or liproxstatin-1 (1 μM) for 24 h, and then intracellular MDA (c), intracellular 8-OHdG (d), extracellular HMGB1 (e), and cell viability (f) were assayed (n= 3 well/group, two way ANOVA with Tukey's multiple comparisons test on all pairwise combinations). Data in (a-f) are presented as mean ± SD. The results in (a-f)

are representative of those from 2 independent experiments with 3 technical replicates each.

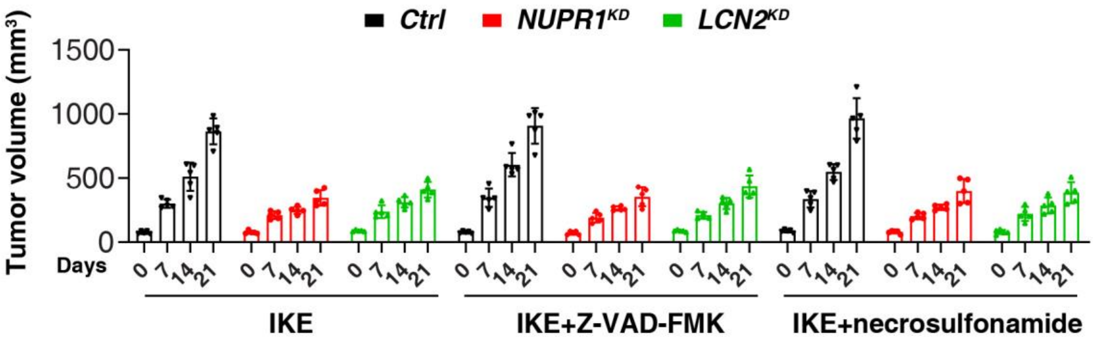

Supplementary Figure 5. Effects of Z-VAD-FMK and necrosulfonamide on IKE-mediated tumor suppression *in vivo*. Athymic nude mice were injected subcutaneously with indicated *PDHA1*-knockdown (*NUPR1*^{KD}) or *LCN2*-knockdown (*LCN2*^{KD}) PANC1 cells for 7 days and then treated with IKE (40 mg/kg, i.p., once every other day) in the absence or presence of Z-VAD-FMK (10 mg/kg, i.p., once every other day) or necrosulfonamide (10 mg/kg, i.p., once every other day) starting at day 7 for 2 weeks. Tumor volumes were calculated weekly (n = 5 mice/group). Data are presented as mean ± SD. The results are representative of those from 2 independent experiments.

Supplementary Table 1

Primers used in this study

Gene	Forward	Reverse
human *NUPR1*	5'-GACTCCAGCCTGGATGAATCTG-3'	5'-CTTCTCTCTTGGTGCGACCTTTC-3'
mouse *Nupr1*	5'-GAATATGATCAGTACAGCCTGGC-3'	5'-CAGAGTTCTGGAACTTGGTCAGC-3'
human *LCN2*	5'-GTGAGCACCAACTACAACCAGC-3'	5'-GTTCCGAAGTCAGCTCCTTGGT-3'
mouse *Lcn2*	5'-ATGTCACCTCCATCCTGGTCAG-3'	5'-GCCACTTGCACATTGTAGCTCTG-3'
human *PTGS2*	5'-CGGTGAAACTCTGGCTAGACAG-3'	5'-GCAAACCGTAGATGCTCAGGGA-3'
mouse *Ptgs2*	5'-GCGACATACTCAAGCAGGAGCA-3'	5'-AGTGGTAACCGCTCAGGTGTTG-3'
human *ATF4*	5'-TTCTCCAGCGACAAGGCTAAGG-3'	5'-CTCCAACATCCAATCTGTCCCG-3'
mouse *Steap3*	5'-TCTTCAGCACCGCCAGTCTAAC-3'	5'-CTGGCTGATCACTGCAGATGAG-3'
mouse *Tfrc*	5'-GAAGTCCAGTGTGGGAACAGGT-3'	5'-CAACCACTCAGTGGCACCAACA-3'
mouse *Slc11a2*	5'-TTGCAGCGAGACTTGGAGTGGT -3'	5'-GCTGAGCCAATGACTTCCTGCA -3'
mouse *Ftl*	5'-CCTCGAGTTTCAGAACGATCGC-3'	5'-CCTGATTCAGGTTCTTCTCCATG-3'
mouse *Fth1*	5'-GCCGAGAAACTGATGAAGCTGC-3'	5'-GCACACTCCATTGCATTCAGCC-3'
mouse *Slc40a1*	5'-CCATAGTCTCTGTCAGCCTGCT-3'	5'-CTTGCAGCAACTGTGTCACCGT-3'
human *18SRNA*	5’-CTACCACATCCAAGGAAGCA-3’	5’-TTTTTCGTCACTACCTCCCCG-3’
mouse *18srna*	5’-GCAATTATTCCCCATGAACG-3’	5’-GGCCTCACTAAACCATCCAA-3’
