# Supplementary material for: NUPR1 is a critical repressor of ferroptosis
Source: Nat Commun. 2021 Jan 28;12:647. doi: 10.1038/s41467-021-20904-2 (PMC7843652; doi:10.1038/s41467-021-20904-2)
Supplement: Supplementary file 2 — Reporting Summary [file 41467_2021_20904_MOESM2_ESM.pdf]

nature research

Corresponding author(s): Daolin Tang; Guido Kroemer

Last updated by author(s): Nov 21, 2020

Reporting Summary

Nature Research wishes to improve the reproducibility of the work that we publish. This form provides structure for consistency and transparency in reporting. For further information on Nature Research policies, see Authors & Referees and the Editorial Policy Checklist .

Statistics

For all statistical analyses, confirm that the following items are present in the figure legend, table legend, main text, or Methods section.

n/a	Confirmed	
☐	☒	The exact sample size (*n*) for each experimental group/condition, given as a discrete number and unit of measurement
☐	☒	A statement on whether measurements were taken from distinct samples or whether the same sample was measured repeatedly
☐	☒	The statistical test(s) used AND whether they are one- or two-sided<br>*Only common tests should be described solely by name; describe more complex techniques in the Methods section.*
☒	☐	A description of all covariates tested
☒	☐	A description of any assumptions or corrections, such as tests of normality and adjustment for multiple comparisons
☐	☒	A full description of the statistical parameters including central tendency (e.g. means) or other basic estimates (e.g. regression coefficient) AND variation (e.g. standard deviation) or associated estimates of uncertainty (e.g. confidence intervals)
☐	☒	For null hypothesis testing, the test statistic (e.g. *F*, *t*, *r*) with confidence intervals, effect sizes, degrees of freedom and *P* value noted<br>*Give P values as exact values whenever suitable.*
☒	☐	For Bayesian analysis, information on the choice of priors and Markov chain Monte Carlo settings
☒	☐	For hierarchical and complex designs, identification of the appropriate level for tests and full reporting of outcomes
☐	☒	Estimates of effect sizes (e.g. Cohen's *d*, Pearson's *r*), indicating how they were calculated

Our web collection on statistics for biologists contains articles on many of the points above.

Software and code

Policy information about availability of computer code

Data collection	GraphPad Prism 8.4.3 was used to collect and analyze data. NanoString results were produced from RCC files using nSolver Analysis Software 3.0.
Data analysis	GraphPad Prism 8.4.3 was used to collect and analyze data. NanoString results were produced from RCC files using nSolver Analysis Software 3.0. Q-PCR analysis was performed using Bio-Rad CFX Manager software 3.1. GEPIA (http://gepia.cancer-pku.cn/index.html), an interactive web server for analyzing the TCGA data, was used to separate the TCGA cohorts.

For manuscripts utilizing custom algorithms or software that are central to the research but not yet described in published literature, software must be made available to editors/reviewers. We strongly encourage code deposition in a community repository (e.g. GitHub). See the Nature Research guidelines for submitting code & software for further information.

Data

Policy information about availability of data

All manuscripts must include a data availability statement. This statement should provide the following information, where applicable:

- Accession codes, unique identifiers, or web links for publicly available datasets
- A list of figures that have associated raw data
- A description of any restrictions on data availability

All the other data supporting the findings of this study are available within the article and its supplementary information files and from the corresponding author upon reasonable request. GEPIA (http://gepia.cancer-pku.cn/index.html) was used to analyze the TCGA data of NURP1 (Ensembl ID: ENSG00000176046.8) or LCN2 (Ensembl ID: ENSG00000148346.11) gene. Source data are provided with this paper.

Field-specific reporting

Please select the one below that is the best fit for your research. If you are not sure, read the appropriate sections before making your selection.

- [x] Life sciences
- [ ] Behavioural & social sciences
- [ ] Ecological, evolutionary & environmental sciences

For a reference copy of the document with all sections, see nature.com/documents/nr-reporting-summary-flat.pdf

Life sciences study design

All studies must disclose on these points even when the disclosure is negative.

Sample size	No statistical methods were used to predetermine sample sizes, but our sample sizes (n=10 mice/group) for survival assay are similar to those generally employed in the field (Cell Chemical Biology. 2019, 26: 623–633; World J Gastroenterol. 2003, 9:584-589).
Data exclusions	No data was excluded.
Replication	Replicates in all assays were confirmatory and the extent described within the text and shown in the main figures.
Randomization	Animal experiments were carried out with randomly chosen littermates of the same sex and matched by age and body weight. For in vitro studies, all cells from multiple dishes were combined and then plated into wells that were treated with various drugs. Thus, all treatment groups came from the same cell stock. Biological independent experiments were performed on independent aliquots of cells thawed from the liquid nitrogen freezer.
Blinding	Animal treatments were performed by technicians who were not blind, but not involved in sample measurement. All in vitro experiments were not blind, because postdoctoral fellows had their own independent projects, and it is impossible for others to replace them to treat cells and analyze samples.

Reporting for specific materials, systems and methods

We require information from authors about some types of materials, experimental systems and methods used in many studies. Here, indicate whether each material, system or method listed is relevant to your study. If you are not sure if a list item applies to your research, read the appropriate section before selecting a response.

Materials & experimental systems

n/a	Involved in the study	
	x	Antibodies
	x	Eukaryotic cell lines
x		Palaeontology
	x	Animals and other organisms
x		Human research participants
x		Clinical data

Methods

n/a	Involved in the study	
x		ChIP-seq
x		Flow cytometry
x		MRI-based neuroimaging

Antibodies

Antibodies used	All antibodies were commercial antibodies and were listed in Materials and methods: The antibody to ACTB (3700; RRID:AB_2242334; 1:1000) was obtained from Cell Signaling Technology. The antibody to LCN2 (ab63929; RRID:AB_1140965; 1:500) was obtained from Abcam. The antibody to NUPR1 (SAB2109172 [RRID:AB_2868575; 1:500] or sc-23283 [RRID:AB_2157971; 1:100]) was obtained from Sigma-Aldrich or Santa Cruz Biotechnology. Secondary antibodies: goat anti-rabbit IgG secondary antibody (Cell Signaling Technology, 7074, RRID:AB_2099233, 1:1000); horse anti-mouse IgG secondary antibody (Cell Signaling Technology, 7076, RRID:AB_330924, 1:1000); rabbit anti-goat IgG secondary antibody (Abcam, ab6741, RRID:AB_955424, 1:1000).
Validation	Below are validation statements from manufacturers as well as validation performed in-house and by other investigators.<br>ACTB (#3700, RRID:AB_2242334, Mouse mAb [8H10D10]; WB, https://www.cellsignal.com/products/primary-antibodies/b-actin-8h10d10-mouse-mab/3700);<br>LCN2 (#ab63929, RRID:AB_1140965, Rabbit polyclonal; WB, https://www.abcam.com/lipocalin-2-ngal-antibody-ab63929.html);<br>NUPR1 (#SAB2109172, RRID:AB_2868575, Rabbit polyclonal; WB, https://www.sigmaaldrich.com/catalog/product/sigma/sab2109172?lang=en&region=US);<br>NUPR1 (#sc-23283, RRID:AB_2157971, Goat polyclonal; WB, https://datasheets.scbt.com/sc-23283.pdf);<br>Goat anti-rabbit IgG secondary antibody (#7074, RRID:AB_2099233; WB, https://www.cellsignal.com/products/secondary-antibodies/anti-rabbit-igg-hrp-linked-antibody/7074);<br>Horse anti-mouse IgG secondary antibody (#7076, RRID:AB_330924; WB, https://www.cellsignal.com/products/secondary-

antibodies/anti-mouse-igg-hrp-linked-antibody/7076);
Rabbit anti-goat IgG secondary antibody (#ab6741, RRID:AB_955424; WB, https://www.abcam.com/rabbit-goat-igg-hl-hrp-ab6741.html).

Eukaryotic cell lines

Policy information about cell lines

Cell line source(s)	The PANC1 (CRL-1469) and MIAPaCa2 (CRL-1420) cell lines were obtained from the American Type Culture Collection. PHsPDAC cells were generated as previously described (ref 64,65). WT and Nupr1-/- mPDAC cells were generated from Nupr1 +/+;Pdx1-cre;LSL-KrasG12D or Nupr1−/−;Pdx1-cre;LSL-KrasG12D mice, respectively, which was a gift from Juan Iovanna (Centre de Recherche en Cancérologie de Marseille, INSERM, France). WT and Nupr1-/- mPDAC were used at <10 passages.
Authentication	All cells used were authenticated using STR profiling.
Mycoplasma contamination	Mycoplasma testing was negative.
Commonly misidentified lines (See ICLAC register)	The study is not involved in misidentified lines.

Animals and other organisms

Policy information about studies involving animals; ARRIVE guidelines recommended for reporting animal research

Laboratory animals	Pancreatic-specific Lcn2-knockout mice were generated by crossing floxed Lcn2 (Lcn2flox/flox) and Pdx1-Cre transgenic mice. Lcn2flox/flox mice were a gift from Bin Gao (National Institutes of Health, USA). Pdx1-Cre mice (014647) were purchased from the Jackson Laboratory. All mice were C57BL/6 background. All mice used in the pancreatitis experiment were matched for age and sex (6-8 weeks old; male: female: 1:1).
Wild animals	No wild animals were used in the study.
Field-collected samples	No field collected samples were used in the study.
Ethics oversight	We conducted all animal care and experimentation in accordance with the Association for Assessment and Accreditation of Laboratory Animal Care guidelines (http://www.aaalac.org) and with approval from Institutional Animal Care and Use Committees (Guangzhou Medical University [#2019075] and UT Southwestern Medical Center [#102605]) .

Note that full information on the approval of the study protocol must also be provided in the manuscript.
